# Emotional Prototypicality Ratings for 636 Chinese Words: A Database of Chinese Words with Affective Information

**DOI:** 10.1007/s10936-023-10018-9

**Published:** 2023-09-22

**Authors:** Ruiyao Zheng, Meng Zhang, Taomei Guo, Marc Guasch, Pilar Ferré

**Affiliations:** 1 Department of Psychology and CRAMC, Universitat Rovira i Virgili, Carretera de Valls, s.n., 43007 Tarragona, Spain; 2 School of English Studies, Sichuan International Studies University, Chongqing, China; 3 State Key Laboratory of Cognitive Neuroscience and Learning, Beijing Normal University, Beijing, China

**Keywords:** Emotion words, Prototypicality, Valence, Arousal, Emotionality

## Abstract

Exemplars of concepts vary in their degree of prototypicality. This is also true for emotion concepts. This study presents prototypicality ratings for a large set of Chinese words. The database contains 636 potential Chinese emotion words (i.e., words that directly express particular emotions, like “高兴 *happy*” and “哀愁 *sad*”), from different grammatical categories. Native Chinese speakers rated the words in terms of emotional prototypicality. The database also contains values for valence, arousal, and emotionality. The analyses of the ratings revealed that 502 out of 636 words had a high prototypicality value (value equal to or above three on a 1-to-5 scale), the most prototypical words being negative and high-arousal words. The analyses also indicated that the emotional prototypicality of a word was positively related to both arousal and emotionality, and negatively related to valence. Among these variables, arousal was the most important contributor. Similar results have been found in studies conducted in other languages. This will be a useful resource for researchers interested in studying emotion words in the Chinese language and for those interested in cross-linguistic comparisons.

## Introduction

Emotions are closely linked to human life and are essential for human mental activity. A highly relevant topic in the study of emotions has been their universality, that is, the extent to which there is a set of basic emotions shared by all people. Paul Ekman has been one of the major contributors to this view. He proposed the existence of seven basic emotions (anger, disgust, fear, surprise, happiness, sadness, and contempt), which would occur involuntarily, in response to some stimuli [actual, imagined, or re-experienced stimuli (Ekman, 2004; Ekman & Friesen, 1971; Ekman et al., 1987; Russell, 1994]. These emotions could be perceived by all humans, regardless of their culture, ethnicity, language, or geography (Ekman, 1971, 1973; Ortony & Turner, 1990). The study of the universality of emotions has mostly relied on faces (Carroll & Russell, 1996; Masuda et al., 2008; Padgett & Cottrell, 1996; Rutter et al., 2019; Schindler et al., 2019; Small & Verrochi, 2009). The lexicon of emotional words, that is, of words related in some way to human emotions, and the comparison across languages and cultures, can, therefore, also be considered a useful approach in this field.

Different approaches have characterized the affective properties of emotional words and other types of emotional stimuli. One of the most influential is the dimensional approach. From this perspective, emotions can be characterized in terms of two dimensions: valence, and arousal (Mauss & Robinson, 2009). Valence is the level of pleasantness produced by a stimulus, ranging from highly negative to highly positive, while arousal refers to the level of autonomic activation produced by it, ranging from calming to exciting (Bestelmeyer et al., 2017; Feldman, 1995; Kensinger, 2004). Therefore, emotional words may be positive or negative and may vary in arousal levels. In relation to that, the pioneering work of Bradley and Lang (1999) has had a broad impact. They collected affective norms for a large set of English words by asking participants to rate them in terms of valence and arousal (and also in relation to dominance, a less studied affective dimension). As a result, they published the *Affective Norms for English Words* (ANEW), which has served as inspiration for a series of studies conducted since then in other languages. There are, indeed, affective norms for Spanish (Duchon et al., 2013; Ferré et al., 2012; Guasch et al., 2016; Redondo et al., 2007; Stadthagen-Gonzalez et al., 2017), European Portuguese (Soares et al., 2012), Italian (Barca et al., 2002; Montefinese et al., 2014), French (Monnier & Syssau, 2014), German (Kanske & Kotz, 2010), Polish (Imbir, 2015), and Chinese (Xu et al., 2022; Yao et al., 2017), among others.

The datasets listed above contain large sets of words characterized by their valence and arousal. Part of them may be considered emotional words (that is, related to emotions in some way). Pavlenko (2008) pointed out that a relevant distinction should be made within emotional words. Concretely, “emotion words” (EM words henceforth) are those that directly express a particular emotion, such as “happy” and “sad.” In contrast, “emotion-laden words" (EL words henceforth) do not denote an emotional state but can, notwithstanding, provoke emotions, including words like “wedding” and “death.” The affective properties of EL words may be more prone to individual and cultural differences than those of EM words (Bromberek-Dyzman et al., 2021; Zhang et al., 2020). The reason is that the emotional content of EL words is, most probably, acquired in relation to emotional experiences. For example, the word “vacation” may elicit a positive feeling in many people, but it might also bring back bad memories for others, eliciting a negative feeling in consequence. In contrast, considering that EM words directly denote emotions, their emotional content is expected to be more stable across speakers. Research on emotional word processing commonly relies on normative studies (i.e., like the ones mentioned in the above paragraph) to select the experimental stimuli. However, these datasets do not distinguish between EM and EL words, so any researcher interested in studying EM words must select them intuitively, without any established objective criterion. This is the case of the few studies examining the differences in processing between EM and EL words (Kazanas & Altarriba, 2015; Wang et al., 2019; Zhang et al., 2017, 2019).

A recent study might contribute to overcoming that limitation. Ng et al. (2019) classified Chinese emotional words into distinct types, relying on the criteria proposed by Pavlenko (2008). Apart from EM words and EL words, Pavlenko considered a third category, the so-called emotion-related words (i.e., words that describe specific behaviors associated with emotions, such as 苍白 ‘pale’ or 颤抖 ‘shiver’). The dataset collected by Ng et al. (2019) is the first one in Chinese to distinguish between categories of emotional words. However, despite its potential utility as a research tool, the list of words has not been made available to the scientific community. In this study, we aim to fill in this gap by providing an extensive list of potential EM Chinese words (i.e., words denoting emotions). To that end, we have relied on an objective measure: the emotional prototypicality of words.

A prototype can be defined as the most evident case or best example of a particular category (Fehr, 1988; Rosch, 1973). For example, when it comes to means of transport, most people think of cars, which would be considered a prototypical example of that category. The prototype approach, popularized by Rosch (2002), has also been applied to study emotion concepts, with the rationale that, as happens in other categories, there are more and less representative exemplars of the “emotion” concept. In studies conducted within this framework, participants are provided with a set of potential emotion words and are asked to rate the degree to which each refers to an emotion (e.g., Pérez-Sánchez et al., 2021). A pioneering study in this line was that of Fehr and Russell (1984), in which participants rated 20 target emotion words (e.g., love, sadness, and hate) on a scale ranging from 1 to 6 (1 = extremely poor example of emotion; 6 = extremely good example of emotion). Similarly, Shaver et al. (1987) provided ratings for 213 English emotion words, finding that most of them scored high on emotional prototypicality.

A few studies inspired by the work of Fehr and Russell (1984) and Shaver et al. (1987) have been published over the course of the last two decades. For example, Zammuner (1998) selected a set of potential emotion terms in Italian (153) and collected ratings for a series of affective variables to know which contributed most to emotional prototypicality. These variables were valence (of note, the concept of valence here was not the same as that of dimensional models, because it refers to the degree to which the word denotes an emotional experience, regardless of its positive or negative polarity), intensity (a concept similar to arousal, but also including other components of emotional impact, such as peak intensity, or onset latency), and duration (the length of time the emotional experience is maintained). This study showed that the three variables predicted prototypicality ratings, and intensity was the one with higher predictive capacity. Furthermore, the relationship with emotional prototypicality was positive for both valence and intensity and negative for duration. Considering these results, Zammuner (1998) concluded that the more prototypical words were those with more extreme valence and intensity values as well as those denoting a brief affective experience. Using the same approach, Niedenthal et al. (2004) conducted an emotional prototypicality rating study in French. In addition to valence and intensity, frequency and age of acquisition were included as possible predictors in their analysis. The results were similar to those of Zammuner (1998). That is, both intensity and valence predicted prototypicality (i.e., high scores in intensity and valence predicted high scores in emotional prototypicality), with intensity being the more predictive variable. A more recent, and altogether larger, study in this line of research is the one published by Pérez-Sánchez et al. (2021). These authors collected emotional prototypicality ratings for 1286 potential Spanish emotion words. They also provided the ratings for those words in other affective (valence, arousal, emotionality, happiness, sadness, fear, disgust, and anger), and psycholinguistic (age-of-acquisition, frequency, and concreteness) variables. In their study, the variable which best predicted prototypicality was emotionality (a variable that describes the extent to which a word has an emotional charge, regardless of its positive or negative polarity). Consequently, words with a higher emotional charge tended to be those with higher prototypicality scores. The study also successfully identified sadness, happiness, anger, and arousal as positive predictors of prototypicality, while disgust, age of acquisition and frequency were found to act as negative predictors. Therefore, the most prototypical emotion words can be described as highly arousing words, related to the emotions of sadness, happiness, and anger. Furthermore, they tend to be low frequency words, which were acquired at an early stage in development. Finally, the authors also explored the role of grammatical category on emotional prototypicality, bringing out a clear preponderance of adjectives over nouns and verbs among the highly prototypical words of the database (i.e., those with a prototypicality rating equal or above 3 on a 1–5 scale).

The main aim of this study was to provide normative data on the emotional prototypicality of a large set of Chinese words, in accordance with the procedure followed by Pérez-Sánchez et al. (2021). Although a few previous studies include affective ratings for Chinese words (e.g., Xu et al., 2022; Yao et al., 2017), these do not distinguish between EM and EL words. As mentioned previously, the only study that has made such a distinction (Ng et al., 2019) does not feature a readily available list of words. The second aim of this study was to explore the contribution of several factors to emotional prototypicality, in particular, valence, arousal, and emotionality. Emotionality was defined, much as it was by Pérez-Sánchez et al. (2021), as the emotional load of a word, regardless of its polarity. To these ends, we selected 636 potential EM words and collected emotional prototypicality ratings from native speakers of Mandarin. With respect to valence and arousal values, we relied on the database of Xu et al. (2022) and collected ratings through questionnaires for the words not included in that study. To the best of our knowledge, this is the first database of Chinese emotion words elaborated from an emotional prototypicality approach. It will be a valuable resource for researchers interested in these words per se or their comparison with EL words.

## Method

### Participants

Participants were students from Beijing Normal University and Sichuan International Studies University. After data cleaning (see the Results section), the responses of 261 participants were used for analyses. The mean age of these participants was 20.1 years (range, 17–29; SD*:* 2.3), and 211 (80.8%) were women. All participants were native speakers of Mandarin and came from different regions of China: Northeast China (6), North China (72), East China (32), Northwest China (1), Southwest China (116), Central and Southern China (34). They participated as volunteers and signed an informed consent document.

### Materials

We selected a set of 636 potential EM words from previous normative studies and from studies about emotional word processing (Chen et al., 2015; Lin & Yao, 2016; Wang et al., 2019; Xu et al., 2022; Yao et al., 2017; Zhou & Tse, 2020). To that end, we relied on the criteria outlined by Pavlenko (2008), that is, we selected words directly expressing affective states, like “angry” or “pleasure.” This selection was done by two of the authors who are native speakers of Chinese. The two judges agreed that the final 636 stimuli were potential EM words. The words had 1 to 4 characters. Concretely, there were 13 one-character words (2%), 575 two-character words (90.4%), 14 three-character words (2.2%), and 34 four-character words (5.3%). In relation to grammatical category, there were 348 adjectives (57.4%), 48 nouns (7.5%), and 240 verbs (37.7%).

### Procedure

We collected emotional prototypicality ratings for the 636 words included in the study. Accordingly, words were randomly divided into four questionnaires, with 159 words per questionnaire. Firstly, participants provided sociodemographic data. Secondly, they were asked about their age, gender, and native language. Finally, were also asked if they had lived in China for the last seven years and whereabouts they had lived. After that, participants were presented with the instructions for the rating task. The task instructions were taken from Pérez-Sánchez et al. (2021) and translated to Chinese. Participants were presented with 159 words and asked to rate the extent to which each word referred to an emotion. The rating was done on a scale ranging from 1 = *“这个词语没有任何感情色彩/这个词并不是指一种情绪”* (This word does not refer to an emotion) to 5 = *“这个词有非常强烈的感情色彩/这个词显然指的是一种情感”* (This word clearly refers to an emotion).

In addition to the aforementioned, we created a series of questionnaires to collect valence and arousal ratings for 264 words that were not included in the database of Xu et al. (2022). In these questionnaires, we also included a set of filler words already rated by Xu et al. (2022), concretely, 40 neutral words, and 30 EL words. The reason was to provide participants with a more diverse set of words to rate (note that there were only potential EM words in our dataset, so it might have been strange for participants to rate valence and arousal of a list including only words denoting emotions). Overall, 334 words (264 target words and 70 filler words) were rated in terms of valence and arousal, which were distributed in 6 questionnaires (3 for valence and 3 for arousal, with 111–112 words each). We used the same scale and labels as Xu et al. (2022). Participants were first asked to fill in the same demographic information as that included in the emotional prototypicality questionnaires. Then they were required to rate either the valence of each word on a 7-point scale (from − 3 to + 3, − 3 = extremely negative, 0 = “neutral,” + 3 = extremely positive) or its arousal on a 5-point scale (from 0 to 4, 0 = very low arousal, 4 = very high arousal). The full instructions (emotional prototypicality, valence, and arousal) are provided in the Appendix.

For all the questionnaires and variables, a list of 15–20 words was presented on each screen with a rating scale under each word. The presentation order of the words was randomized in each questionnaire. Participants were instructed to select the option “I don’t know the word” for cases in which they were not familiar with the meaning of a particular word. All the participants rated only one variable and completed a single questionnaire. The questionnaires were all completed online.

## Results

### Data Trimming

The questionnaires were submitted to a trimming procedure to discard non-native speakers of Chinese and participants who had responded with anomalous patterns. The motives of exclusion were the following: participants who rated more than 95% of the words in a questionnaire with the same value; participants who marked the option “I don’t know the word” in more than 50% of the words, and participants whose ratings correlated less than 0.10 with the average rating of the other participants.

As a result of the above exclusion criteria, a total of 25 participants were removed and 261 participants were kept. After cleaning, each questionnaire was completed by an average number of 26.10 participants (SD = 3.28), with the minimum number of participants per questionnaire being 21 and the maximum 30.

### Description of the Database

The database is available at https://figshare.com/articles/dataset/prototypicality_dataset_xlsx/20209859

**Fig. 1:**
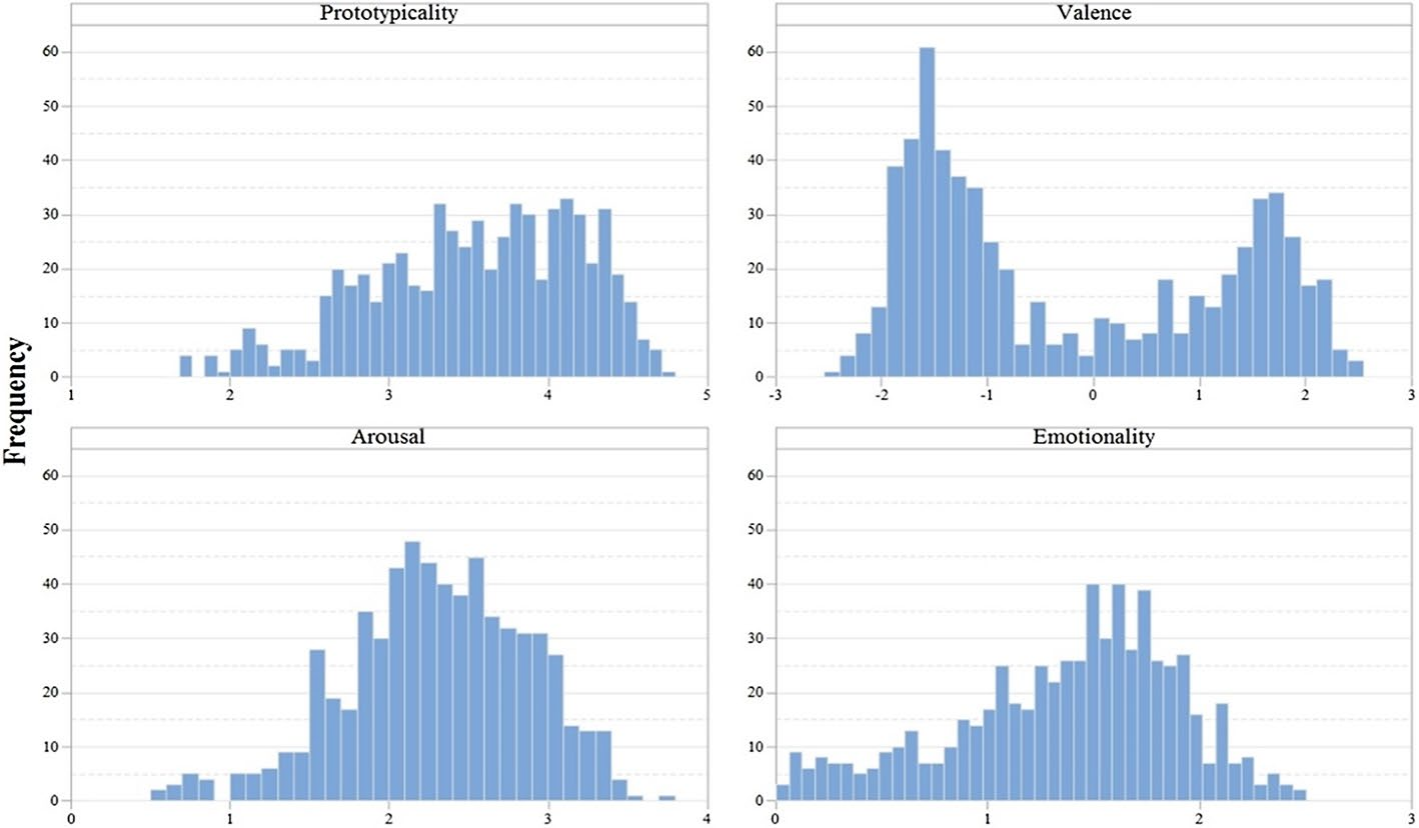
Histograms of prototypicality, valence, arousal, and emotionality

Descriptive statistics and the histograms for all the variables are shown in Table 1 and Fig. 1. Prototypicality data are based on the collected ratings for all the words (i.e., 636 words). Valence and arousal data are based on the collected ratings for part of the words (264 words) and Xu et al.’s (2022) ratings for the other words (372 words). Emotionality was operationalized as valence in absolute terms. To show just an example, “悲痛 grieved” is a negative word with a valence value of − 1.96. Therefore, its emotionality score would be 1.96. The emotionality of “喜悦 joy,” a positive word with a valence value of + 1.96, would be exactly the same (i.e., 1.96). Both words have the same amount of emotional charge, although in one case the charge is positive, while in the other it is negative.

**Table 1:** Descriptive statistics of the full dataset and the high prototypicality subset of words in all the variables

Variable	Mean	SD	Median	Min	Max
	*Full set*				
Prototypicality	3.54	0.66	3.60	1.70	4.76
Valence	− 0.22	1.46	− 0.85	− 2.42	2.48
Arousal	2.30	0.58	2.31	0.50	3.47
Emotionality	1.37	0.53	1.48	0.00	2.48
	*High prototypicality subset*				
Prototypicality	3.80	0.45	3.79	3.00	4.76
Valence	− 0.36	1.47	− 1.08	− 2.42	2.48
Arousal	2.36	0.54	2.36	0.50	3.74
Emotionality	1.43	0.51	1.52	0.04	2.48

As shown in Fig. 1, there are many words with prototypicality ratings above 3. (502 words, 78.93% of the entire set). Furthermore, the distribution of the words in the valence variable shows two peaks, with a higher concentration of words in the first peak (around a valence value of − 1.5) than in the second peak (around a valence value of + 1.5). Nevertheless, the distribution of arousal and emotionality tends to be normal, although emotionality shows a moderate negative skew. The median values of arousal and emotionality (2.31 and 1.48, respectively) are very close to the scales’ midpoint (2 and 1.5, respectively). Overall, the distribution of the words in the different variables indicates that most of them are negative words (the mean valence value is lower than 0), have a mid-to-high arousal value (the mean value is higher than 2), are mildly emotional (the mean value is close to 1.5) and have a high emotional prototypicality (the mean value is higher than 3).

### Reliability and Validity of the Ratings

We examined the inter-rater reliability of the ratings with a split-half procedure, using the *splithalf.r* function set to 100 repetitions in the *multicon* package (Sherman & Serfass, 2015) in R (v4.2.2, R Core Team, 2022) and RStudio (v2023.6.1.524, Rstudio Team, 2023). There were several versions of each questionnaire (four versions for prototypicality questionnaires, three versions for valence questionnaires, and three versions for arousal questionnaires). We randomly split the participants in each questionnaire into two equal groups and ran a Pearson correlation with the mean ratings of the words of each group. Then, we repeated these steps 100 times to get an average by applying the Spearman-Brown correction. This was repeated for each variable and averaged across questionnaires. The mean correlations obtained indicated a high level of internal consistency for the three variables: prototypicality, *r* = 0.91 [range of 0.87–0.94]; valence, *r* = 0.99 [range of 0.98–0.99]; arousal, *r* = 0.94 [range of 0.90–0.97].

We also examined the validity of our ratings. To our knowledge, there are no previous studies on emotional prototypicality in Chinese. Therefore, we compared our ratings to those obtained in studies conducted in other languages. To that end, we focused on the words in common with these studies (after translating them). To obtain the translation equivalents between the Chinese words and the words in other languages, we relied on the following sources: for Spanish translations, we used Esdict. (n.d.); for English translations, we relied on the Harper Collins Publishers Ltd. (2014); for the French and Italian translations, we employed the online translation website DeepL Translator. (n.d.). In all cases, we followed a two-steps procedure: firstly, we translated the Chinese word to the target language (e.g., English). Then, we back translated that word to Chinese. We kept, for the purpose of validity analyses, only the words matching in the two directions of the translation. To examine validity, we computed the correlation between the ratings of the translation equivalents across languages. The results of the correlations were as follows: Spanish (Pérez-Sánchez et al., 2021), *r*(296) = 0.632, *p* < 0.01; English (Shaver et al., 1987, Study 1), *r*(130) = 0.600,* p* < 0.01; Italian (Zammuner, 1998), *r*(119) = 0.534, *p* < 0.01; French (Niedenthal et al., 2004), *r*(108) = 0.585, *p* < 0.01.

We also assessed the validity of valence and arousal ratings, by correlating them with those obtained from previous studies. Firstly, we focused on the filler words of our study, which we obtained from Xu et al. (2022). The correlation between both datasets was *r*(70) = 0.979,* p* < 0.01 for valence and *r*(70) = 0.946,* p* < 0.01 for arousal. Secondly, we identified the words in common with other normative studies in Chinese. In the comparison with Yu et al. (2016), we obtained a correlation of *r*(441) = 0.900, *p* < 0.01 for valence and a correlation of *r*(441) = 0.578, *p* < 0.01 for arousal. Regarding the study of Yao et al. (2017), the correlation was of *r*(70) = 0.849, *p* < 0.01 for valence and of *r*(70) = 0.423, *p* < 0.01 for arousal. Lastly, the comparison with the study of Zhou and Tse (2020) revealed a correlation of *r*(110) = 0.901, *p* < 0.01 for valence and a correlation of *r*(110) = 0.474, *p* < 0.01 for arousal.

### Characteristics of the More Prototypical Words

To explore the characteristics of the more prototypical words, we focused on the high-prototypicality subset (i.e., words with an emotional prototypicality rating equal to or above 3). The descriptive statistics for all the variables are presented in Table 1.

Then, we categorized the words of this subset regarding valence and arousal into “negative/positive” words, and into “low-arousal/high-arousal” words, following the criteria used by Xu et al. (2022). Concretely, we categorized words with a valence value under 0 as negative words, and words with a valence value above 0 as positive words. Words with a valence value equal to 0 should be considered as neutral words, although there were no words with this value in the subset of highly prototypical words. Regarding arousal, words with an arousal rating below or equal to 2 were classified as low-arousal words, while those with a value higher than two were classified as high-arousal words. Apart from that, words in the high prototypicality subset were classified according to their part of speech. Among the 502 high-prototypicality words, there were 317 negative words (63.15%) and 185 positive words (36.85%). Furthermore, there were 371 high-arousal words (73.30%) and 131 low-arousal words (26.70%). Regarding the part of speech or grammatical type, there were 285 adjectives (56.77%), 195 verbs (38.65%), and 22 nouns (4.58%). Chi-square tests showed that highly prototypical words are mostly negative (*X*^2^(1, *n* = 502) = 34.71,* p* < 0.001), have a high arousal (*X*^2^(1, *n* = 502) = 114.74, *p* < 0.001), and are, for the most part, adjectives (*X*^2^(2, *n* = 502) = 213.54, *p* < 0.001). Finally, we identified the 15 most prototypical words in the dataset. Their characteristics are shown in Table 2. In this subset, there are ten negative words and five positive words. All of them are high-arousal words and have a large proportion of adjectives between them.

**Table 2:** Affective and psycholinguistic characteristics of the top 15 emotion prototypical words

Word	Translation	Prototypicality	Valence	Arousal	Emotionality	POS*
悲痛	Painfully sad	4.76	− 1.96	2.73	1.96	ADJ
高兴	Happy	4.71	2.39	3.24	2.39	ADJ
悲怆	Pathetic	4.67	− 1.92	2.40	1.92	ADJ
仇视	Regard as an enemy	4.67	− 2.08	2.96	2.08	Verb
愤恨	Indignantly resent	4.67	− 1.44	3.93	1.44	Verb
欣喜	Joyful	4.67	1.80	2.70	1.80	ADJ
忌恨	Envy and hate	4.62	− 1.70	2.05	1.70	Verb
哀痛	Feel the anguish of sorrow	4.60	− 1.75	2.55	1.75	ADJ
喜悦	Charmed	4.60	1.96	2.27	1.96	ADJ
兴高采烈	Elated	4.60	2.11	2.20	2.11	ADJ
义愤填膺	Be filled with indignation	4.60	0.36	3.30	0.36	ADJ
悲愁	Grieve over someone's death	4.57	− 1.48	2.14	1.48	ADJ
暴怒	Violently rage	4.57	− 1.78	3.09	1.78	Verb
盛怒	Very rage	4.55	− 1.75	3.21	1.75	Verb
忿恨	Very resent	4.55	− 1.68	2.83	1.68	Verb

*Part of speech

### Bivariate Relationships between the Affective Variables

We computed the Pearson correlations between all variables for the entire set of words, as can be seen in Table 3. The most closely related variable to emotional prototypicality was arousal. The correlation coefficient between the two variables was 0.315, a value which indicates the presence of a moderate relationship, suggesting that words high in emotional prototypicality tend to have a high level of arousal. Emotional prototypicality was also significantly correlated with the other affective variables. There was a negative correlation with valence, *r* = − 0.288, *p* < 0.001 and a positive correlation with emotionality, *r* = 0.188, *p* < 0.001. These results indicate, therefore, that highly prototypical words tend to be both negative and affectively loaded.

**Table 3:** Correlation between variables

Variable	Prototypicality	Valence	Arousal	Emotionality
Prototypicality	1	− 0.288*	0.315*	0.237*
Valence	–	1	− 0.021	0.021
Arousal	–	–	1	0.188*
Emotionality	–	–	–	1

*Correlation is significant at the level of 0.001 (2-tailed)

### Prediction of Prototypicality

We conducted a multiple linear regression analysis with the entire set of words to examine the predictive capacity of the assessed variables on emotional prototypicality. We first examined the data to detect possible multicollinearity effects between the analyzed variables. Acceptable multicollinearity coefficients were observed: the lowest tolerance value obtained was 0.96, while the highest VIF value obtained was 1.04.

The result of the regression model was significant, *F*(3, 632) = 57.396, *p* < 0.001, *R*^2^ = 0.214 (an adjusted *R*^2^ of 0.210), and the three factors were included in the model (see Table 4). The higher significant standardized Beta coefficient was for the valence variable, followed by arousal and emotionality. This means that all three variables contribute to prototypicality.

**Table 4:** Linear regression analysis on prototypicality

Predictors	*R*^2^ change	Standardized Coefficient beta	*t* value
Arousal	0.099	0.273	7.589*
Valence	0.079	− 0.287	8.129*
Emotionality	0.036	0.192	5.346*

The order of the variables presented here is consistent with the order of their entry in the regression model

*The *t* value is significant at the level of 0.001 (2-tailed)

## Discussion

The present study had two goals. Firstly, we aimed to provide normative data on the emotional prototypicality of a large set of Chinese words. Secondly, we aimed to examine the affective variables which contribute the most to prototypicality.

In relation to the first objective, this study presents ratings of emotional prototypicality for 636 words and ratings for valence and arousal for words not previously included in normative studies. The norms show high indices of reliability for prototypicality, valence, and arousal, thus indicating a high level of internal consistency (Hedge et al., 2018; Parsons et al., 2019). These results are in alignment with the excellent reliability coefficients shown in Pérez-Sánchez et al. (2021) with regard to emotional prototypicality. Our results are also congruent with previous normative studies in terms of the high reliability of the data collected. As is the case with affective and concreteness ratings from Ferré et al. (2017) and Guasch et al. (2016), affective ratings from Xu et al. (2022), and affective, concreteness, familiarity, and imageability ratings from Yao et al. (2017).

With reference to validity, we compared our prototypicality ratings of our words with those of their translation equivalents in other languages. Pearson correlations were middle to high in all the cases (range = 0.53–0.64), the highest correlation being the one obtained in comparison with the Spanish data (Pérez-Sánchez et al., 2021). Our correlation values are reminiscent of those reported in previous studies. For example, Pérez-Sánchez et al. (2021) found moderate to high correlations between the prototypicality ratings of Spanish words and those of their translation equivalents in American English (Shaver et al., 1987), Basque (Alonso-Arbiol et al., 2006), French (Niedenthal et al., 2004), and Italian (Zammuner, 1998), with correlation coefficients ranging from 0.49 to 0.63. Thus, our study aligns with previous studies on emotional prototypicality, and provides further evidence indicating that normative ratings in Chinese are similar to those obtained in other languages.

In addition to prototypicality, we also compared the ratings of valence and arousal with those present in other Chinese normative studies (Yao et al., 2017; Yu et al., 2016; Zhou & Tse, 2020). Pearson correlations were very high for valence (range = 0.84–0.91). However, when it came to arousal, there were mostly moderate correlations [comparisons with Yu et al., (2016), *r*(441) = 0.578; with Yao et al., (2017), *r*(70) = 0.423; with Zhou and Tse (2020), *r*(110) = 0.474]. The lower level of congruence for arousal ratings than for valence ratings is, however, consistent with several studies [Leveau et al., 2012; Pérez-Sánchez et al., 2021, but see Guasch et al., (2016) and Xu et al., (2022), for high validity coefficients for arousal]. This result might be caused by individual differences either in the concept of valence and arousal or in the role of these variables in the affective experience (Barrett, 1998; Feldman, 1995; Kuppens, 2008). Firstly, and according to Pérez-Sánchez et al. (2021), the cause of the lower correlation for arousal (with respect to valence) may be that the concept of arousal is more difficult to understand than the concept of valence. Some participants may conflate both concepts and consider arousal as the intensity of the negative or the positive experience. Other participants, in contrast, may understand arousal as a concept differing from that of valence, which thus indicates the degree of activation produced by the affective experience (Kron et al., 2015). Secondly, the experience of arousal may show greater individual variability than the experience of valence. Indeed, Kuppens (2008) reported individual differences in the extent to which high/low arousal is experienced as pleasant or unpleasant. In relation to this, it should be noted that in some of the first studies in the field (Niedenthal et al., 2004; Zammuner, 1998), the term “intensity” was used instead of “arousal.” The concept of intensity was understood as a broader dimension, including other components (e.g., peak intensity and onset latency). It might be easier for participants to understand what intensity means (and to rate it) than arousal. Further work in addressing this issue may be necessary.

With respect to the characteristics of the high emotion prototypicality words in the dataset (i.e., those with a prototypicality value equal to or above 3), they mostly correspond to negative words with a high level of arousal. There is also a preponderance for adjectives, and this larger number of adjectives among words scoring high on emotion prototypicality is in line with Pérez-Sánchez et al. (2021) and suggests that it may be more adequate to select adjectives rather than nouns in studies focusing on the emotion lexicon (e.g., word processing studies). The other result in agreement with previous findings (e.g., Pérez-Sánchez et al., 2021; Zammuner, 1998) is the larger number of negative emotion words than positive emotion words. One possible explanation is that negative experiences are more differentiated than positive experiences. Among the seven basic emotions proposed by Ekman (1973)-anger, disgust, fear, surprise, happiness, sadness, and contempt-there is only one positive emotion (i.e., happiness). This might be related to the distinct processing styles associated with positive and negative experiences. Indeed, it has been proposed that unpleasant experiences indicate the presence of a problem (e.g., Schwarz & Bless, 1991). Therefore, these experiences elicit an in-depth cognitive analysis of the situation with the objective being to find a suitable solution. This would result in more differentiated labels for those experiences than for positive experiences. In such a case, cognitive processing would be more general (i.e., less detailed) as positive situations indicate the absence of problems and, therefore, do not require a detailed level of processing.

As well as providing an emotional prototypicality dataset, our second aim was to explore the contribution of several affective variables to prototypicality. We found that the three examined variables, that is, valence, arousal, and emotionality, were correlated with emotional prototypicality, arousal being the variable with the highest correlation coefficient. Our results are mostly in concordance with Zammuner (1998) and Niedenthal et al. (2004), both of whom found the strongest correlations for intensity (a factor akin to arousal, but with a broader scope, as explained in the introduction) and who also reported significant correlations between prototypicality and emotionality (labeled as valence in their studies). The results do not entirely match, however, with those of Pérez-Sánchez et al. (2021). Although they, much like us, found significant correlations between the three affective variables, the variable most correlated with prototypicality was emotionality. The regression analysis in our study mostly agreed with the correlation analysis, showing that the three affective variables predicted prototypicality. Therefore, highly emotion prototypical words are mostly negative words, highly arousing, and with extreme valence values. Pérez-Sánchez et al. (2021) also identified emotionality and arousal as relevant predictors of emotional prototypicality. However, these authors included more predictors than us in the regression analysis and as such the results cannot be directly compared across studies.

Finally, it is necessary to name some limitations of the study. First of all, we did not restrict the selection of stimuli to Chinese words with a certain character number, as in other normative studies in this language (e.g., Yao et al., 2017). Neither did we restrict the part of speech of the words included in the dataset, a detail in contrast with studies only using nouns (e.g., Shaver et al., 1987). Although these decisions may make it difficult to carry out comparisons across studies, they were made with the objective of covering as many potential emotion Chinese words as possible. The second limitation is the presence of a gender bias in the sample, with 80.8% of female participants. It must be said that this is a common limitation in normative studies collecting subjective ratings for words. The reason being that, in most cases, participants are university students where women are more prevalent. It should be noted, however, that a high correlation between men’s and women’s affective ratings has been reported (e.g., Montefinese et al., 2014). Future research should strive to include more balanced samples of participants with regard to gender and include subjects from populations other than academia.

## Conclusions

This is a normative study, which includes emotional prototypicality ratings, as well as valence and arousal ratings for six hundred and thirty-six Chinese words. The high emotion prototypicality words are mostly negative words with high-arousal, and adjectives. Arousal, valence, and emotionality contribute to the emotional prototypicality of words with arousal being the most important predictor. The present dataset provides researchers with a list of words characterized by affective properties that can be used to select experimental materials. These words can be used in studies with an emphasis on emotion words per se (e.g., studies about the cognitive and neural processing of these words), as well as in others aimed at making comparisons between emotion words and emotion-laden words.

## Appendix

### Instructions for Prototypicality Rating in Chinese

原型判断.

接下来你会看到一系列的词。我们希望您可以回答以下与这些词有关的问题:

您是否认为这个词指向某一种情感?/判断这个词所代表的感情色彩浓烈的程度.

请在回答这个问题时使用如下1-5的评分标准:

1 = “这个词语没有任何感情色彩/这个词并不是指一种情绪 “

5 = “这个词有非常强烈的感情色彩/这个词显然指的是一种情感 “

根据您自身的情况, 请使用 “1–5 “中的任意数字进行作答。如果您不知道某一词语, 请选择”我不知道这个词语 “。

请记住您的回答没有对错之分, 因此请不要花费太多时间考虑每一个词。

### Instructions for Prototypicality Rating in English

PROTOTYPICALITY.

Next, you will see a series of words. We ask you to please answer the following question for each of them:

To what extent does this word refer to an emotion?

Please respond to all the words in the questionnaire giving your rating on a scale of 1 to 5, where:

1 = “This word does not refer to an emotion”

5 = “This word clearly refers to an emotion”

You can also use any intermediate number on the scale according to what corresponds. If you do not know a word, choose the option “I DON’T KNOW THE WORD”.

Please do not spend too much time thinking about each word and remember that there are no right or wrong responses.

### Instructions for Valence Ratings in Chinese

愉悦度

您的任务是评估一组词语的愉悦度。愉悦度是指阅读该词语让您所产生的积极/愉快或消极/不愉快的感觉。

为了让您更好的理解本次调查, 我们在这里提供两个样例, 向您解释什么是词的愉悦度。例如, “金牌”这个词带有积极情绪, 可能会让您感到愉悦; 而“犯罪”这个词带有消极情绪, 可能会让您感到害怕。

词的愉悦度可以用一个特定的分值区间进行评估。

本次调查的评估采用7分量表, 分值范围为-3- + 3。请根据以下标准, 利用这些分值对每一个单词的**愉悦度**进行评估: 如果这个词让你感觉非常悲伤, 请你给它打-3分; 如果它让你感觉非常快乐, 请你给它打 + 3分; 当然你可以使用其他的分值 (− 2, − 1, + 1, + 2) 来评估你的快乐或悲伤程度。

请对所有词语进行作答。如果您不知道某个特定的词, 请选择 "我不知道这个词 "。

请记住, 答案没有对错之分。您应该根据您看到这个词时的第一反应做出快速评估。

### Instructions for Valence Ratings in English

#### Pleasantness

Your task is to evaluate the pleasantness of a set of words. Pleasantness refers to the positive/pleasant or negative/unpleasant feeling produced by words.

To give just some examples, a positive word may be 金牌 “gold medal”, while a negative word may be 勾当 “criminal dealing”.

Word pleasantness varies on a continuum, with some words falling between the two extremes.

You are asked to perform your ratings on a seven-point scale provided next to each word, where “ − 3” means “extremely negative, “0” means “neutral,” and “+ 3” means “extremely positive.” You can use all the values of the scale.

Please, respond to all the words. In case you don’t know a specific word, you should mark the “I don’t know the word” option.

Remember that there are no right or wrong answers and that you should make a quick assessment based on your first reaction upon seeing the word.

### Instructions for Arousal Ratings in Chinese

唤醒度

您的任务是评估一组词语的唤醒度。唤醒度是指阅读该词语所让您感觉到被激活/唤醒或感觉到平静/放松。

为了让您更好的理解本次调查, 我们在这里提供两个样例, 向您解释什么是词的唤醒度。例如, “台风”这个词是高唤醒度的词, 它可能让你觉得完全被激活 (即非常活跃/被唤醒或非常清醒); 而“文书”这个词是低唤醒度的词, 它可能让你感觉非常平静 (即非常不活跃或非常放松) 。

词的唤醒度可以用一个特定的分值区间进行评估。

本次调查的评估采用5分量表, 分值范围为0-4。请根据以下标准, 利用这些分值对每一个单词的**唤醒度**进行评估: 如果这个词让你感觉非常平静 (即非常不活跃或非常放松), 请你给它打0分; 如果它让你觉得完全被激活 (即非常活跃/被唤醒或非常清醒), 请你给它打4分; 当然你可以使用其他的分值 (1, 2, 3) 来评估您的平静和唤醒程度。

请对所有词语进行作答。如果您不知道某个特定的词, 请选择 "我不知道这个词 "。

请记住, 答案没有对错之分。您应该根据您看到这个词时的第一反应做出快速评估。

### Instructions for Arousal Ratings in English

#### Arousal

Your task is to evaluate the arousal of a set of words. Arousal refers to the activation/arousal or calm/relaxation produced by words.

To give just some examples, a high-arousal word may be 台风 “typhoon” while a low arousal word may be 文书 “paperwork”.

Word arousal varies on a continuum, with some words falling between the two extremes.

You are asked to perform your ratings on a five-point scale provided next to each word, where “0” means “very low arousal” and “4” means “very high arousal.” You can use all the values of the scale.

Please, respond to all the words. In case you don’t know a specific word, you should mark the “I don’t know the word” option.

Remember that there are no right or wrong answers and that you should make a quick assessment based on your first reaction upon seeing the word.
